# WRN conditioned media is sufficient for *in vitro* propagation of intestinal organoids from large farm and small companion animals

**DOI:** 10.1242/bio.021717

**Published:** 2017-03-27

**Authors:** Robin H. Powell, Michael S. Behnke

**Affiliations:** Department of Pathobiological Sciences, School of Veterinary Medicine, Louisiana State University, Baton Rouge, LA 70803, USA

**Keywords:** Organoid, Enteroid, Crypt, Intestine, Farm animal, Companion animal, Conditioned media

## Abstract

Recent years have seen significant developments in the ability to continuously propagate organoids derived from intestinal crypts. These advancements have been applied to mouse and human samples providing models for gastrointestinal tissue development and disease. We adapt these methods for the propagation of intestinal organoids (enteroids) from various large farm and small companion (LF/SC) animals, including cat, dog, cow, horse, pig, sheep and chicken. We show that LF/SC enteroids propagate and expand in L-WRN conditioned media containing signaling factors Wnt3a, R-spondin-3, and Noggin (WRN). Multiple successful isolations were achieved for each species, and the growth of LF/SC enteroids was maintained to high passage number. LF/SC enteroids expressed crypt stem cell marker LGR5 and low levels of mesenchymal marker VIM. Labeling with EdU also showed distinct regions of cell proliferation within the enteroids marking crypt-like regions. The ability to grow and maintain LF/SC enteroid cell lines provides additional models for the study of gastrointestinal developmental biology as well as platforms for the study of host-pathogen interactions between intestinal cells and zoonotic enteric pathogens of medical importance.

## Abstract (precis)

**Summary:** Culturing methods for the propagation of crypt-derived mouse and human enteroids are applicable to large farm and small companion animals, demonstrating conservation of the crypt signaling microenvironment.

## INTRODUCTION

The development of primary cell lines for tissue culture has been an integral foundation to the study of cellular and molecular biology. The long term propagation of tissue culture cell lines works better for certain cell types than others. This is especially true for intestinal epithelial cells which have historically been resistant to expansion in culture, even with immortalization techniques such as SV40 transfection. This problem has largely been solved with the recent development of methods to isolate and expand intestinal crypt cells. Intestinal crypts contain LGR5^+^ stem cells which are the progenitors of the other cell types in the intestinal villus, such as Paneth, goblet and epithelial cells (Biswas et al., 2015; Sato and Clevers, 2013). Three factors, Wnt3a, R-spondin and Noggin (WRN), are required to keep LGR5^+^ cells growing in a stem-like state (Sato et al., 2009). When cultured in a 3D matrix with media containing the WRN factors, mouse intestinal crypt cells expand into organoid structures allowing for the continued propagation of intestinal cells. This has also been shown to be relevant for human intestinal cell lines (Jung et al., 2011; Sato et al., 2011a; VanDussen et al., 2015).

To facilitate the growth and maintenance of intestinal organoids, a mouse fibroblast cell line was engineered to secrete the three WRN factors cloned from murine transcripts, called the L-WRN cell line (Miyoshi et al., 2012; Miyoshi and Stappenbeck, 2013; Willert et al., 2003). Conditioned media (CM) cultured from these cells is effective in growing both mouse and human intestinal crypt organoids, demonstrating its usefulness across diverse organisms (Miyoshi and Stappenbeck, 2013; VanDussen et al., 2015). As intestinal crypt organoids from mouse and human samples currently serve as extensively studied models of development and disease (Clevers, 2016; Dedhia et al., 2016; Foulke-Abel et al., 2014; Jackson and Lu, 2016), we sought to expand the available intestinal organoid (enteroid) cell lines to include other species representing large farm and small companion (LF/SC) animals of veterinary relevance, which are also hosts for various zoonotic pathogens of importance to human health. Enteroids represent the relevant cell types for the growth of enteric pathogens, many of which develop into enteric stages within the terminal ileum of the small intestine, allowing for the possibility of culturing these infectious organisms *in vitro*. This strategy has recently shown great promise with the report of the growth of human noroviruses in human enteroids (Ettayebi et al., 2016). LF/SC enteroids may provide similar platforms for intracellular enteric pathogens such as the growth of *Cryptosporidium parvum* within cow enteroids or the growth of *Toxoplasma gondii* enteric stages within cat enteroids.

## RESULTS AND DISCUSSION

### WRN signaling molecules are conserved in LF/SC animals

**Fig. 1.**
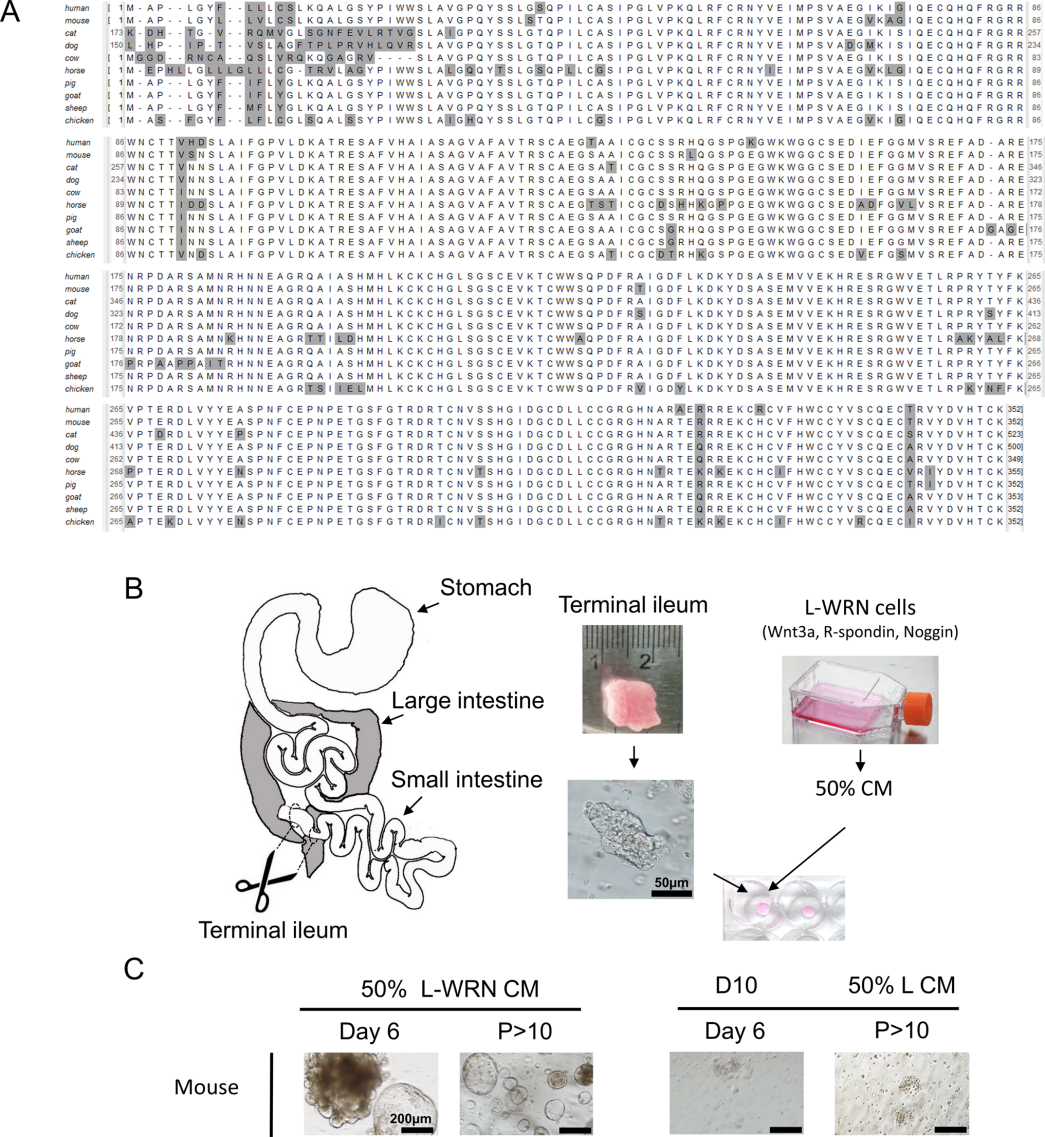
**Conservation of crypt signaling molecules suggests mouse intestine enteroid culture may apply to LF/SC animals.** (A) Alignment of Wnt3a protein orthologs from human, mouse, and LF/SC animals shows broad sequence conservation, non-consensus amino acid (gray). The non-consensus N-terminal region of cat and dog Wnt3a are not shown. (B) Schematic of isolation and growth of enteroids. Tissue samples from the terminal ileum were harvested, trimmed to 1 cm^2^, and crypts were isolated with collagenase digestion. Primary media was incubated with the L-WRN cell line which secrete Wnt3a, R-spondin-3 and Noggin to create 50% L-WRN CM. Isolated crypts were suspended in Matrigel matrix and grown in 50% L-WRN CM. (C) Isolation and growth of mouse crypt enteroids demonstrates successful preparation of 50% L-WRN CM from L-WRN cells. Mouse enteroids only grow in 50% L-WRN CM after isolation (Day 6) to high passage (P>10) number. Mouse enteroids fail to grow when cultured in D10 after isolation (Day 6) or when passed to 50% L CM after high passage (P>10) growth in 50% L-WRN CM.

Given the effectiveness of the WRN factors in stimulating the growth of both mouse and human intestinal enteroids, we were determined to test the activity of CM containing these factors on the growth of enteroids from animals of veterinary relevance. Protein alignments of human, mouse and LF/SC animal Wnt3a (Fig. 1A), R-spondin-3 and Noggin (Fig. S1) show broad protein sequence conservation for all three factors. We obtained the L-WRN cell line from the American Type Culture Collection (ATCC) to produce L-WRN cell conditioned media (50% L-WRN CM), and used it to grow intestinal enteroids (Fig. 1B) (Miyoshi and Stappenbeck, 2013). This media was first tested for the growth of mouse intestinal crypt derived enteroids, which rapidly expanded after isolation from the small intestine, eventually to high passage number (*P*>10) (Fig. 1C), confirming the effectiveness of the media. This growth was specific to 50% L-WRN CM as mouse enteroids failed to develop in DMEM+10% FBS (D10) after crypt isolation. Additionally, after being transferred to L cell conditioned media lacking WRN factors (50% L CM), high passage enteroids previously maintained in 50% L-WRN CM lost morphology breaking apart into individual cells and failing to expand with subsequent passage (Fig. 1C).

### Intestinal crypt-derived enteroids from various LF/SC animals grow and expand to high passage

**Fig. 2.**
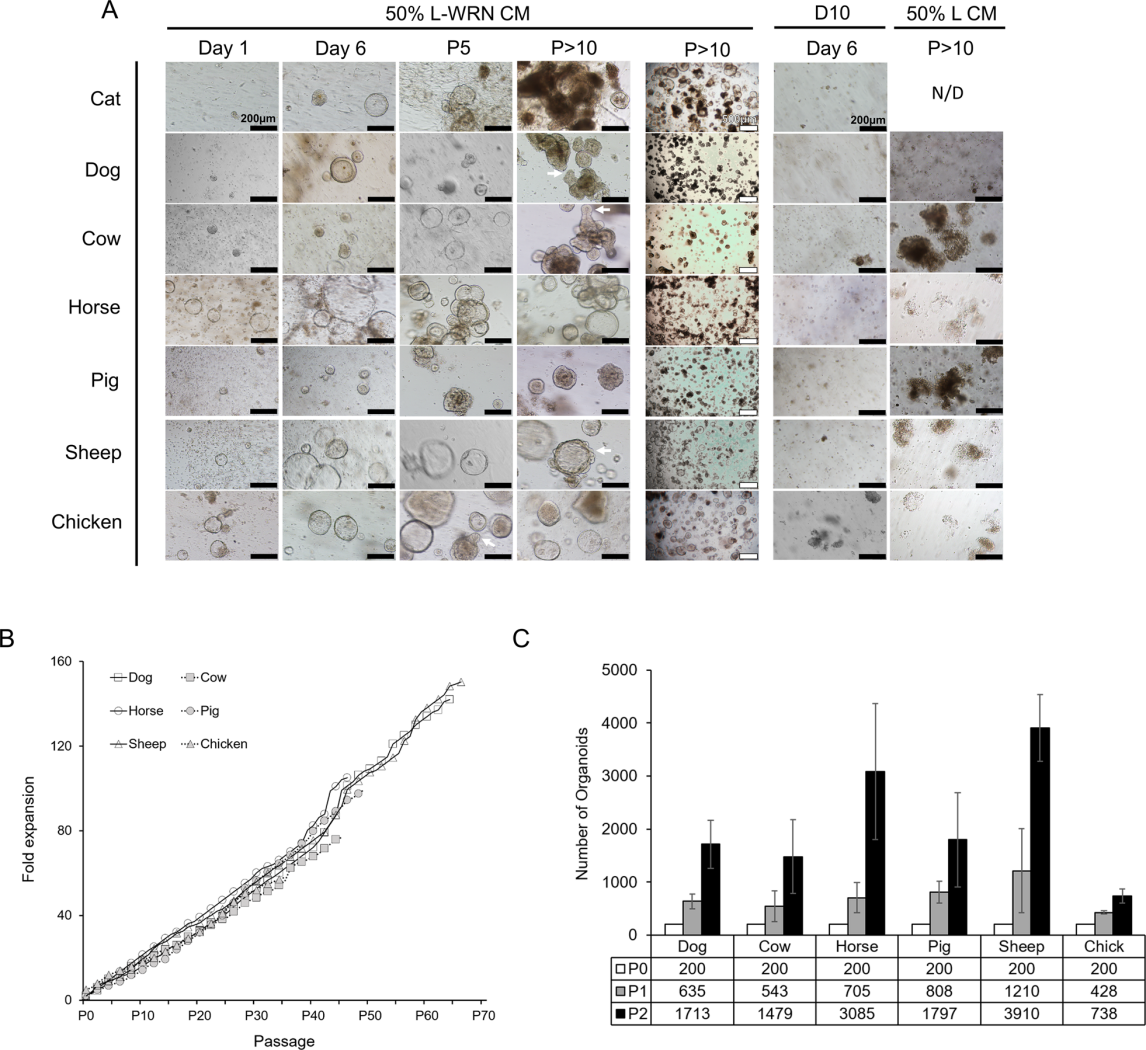
**50% L-WRN CM is required for LF/SC enteroid growth and expansion.** (A) Enteroids from LF/SC animals cultured with 50% L-WRN CM expand soon after crypt isolation (Day 1), growing to large size after passage (Day 6), and continue to expand and obtain crypt-like morphology at higher passage (white arrows) (P5 and P>10). Lower magnification shows enteroids were maintained at high density throughout passage history (P>10, 500 µm). Enteroids fail to grow when cultured in D10 after isolation (Day 6) or when passed to 50% L CM after high passage (P>10) growth in 50% L-WRN CM, no data (N/D). (B) Fold expansion of wells containing LF/SC enteroids. LF/SC enteroids grown in 24-well plates were maintained at high density. The ratio of per well expansion was recorded at each passage throughout the passage history. (C) Number of enteroids expands with each passage. A total of 200 high passage (P>10) enteroids were seeded to two wells and the number of enteroids was counted after 3-4 days of growth, and again after subsequent passage splitting 1:2, *n*=3. Cat samples are not included in B or C due to the lower passage and density of enteroids. Error bars indicate ±s.d.

We began working relationships with local animal processing and shelter facilities to obtain tissue samples from the terminal ileum of the small intestine from a variety of LF/SC animals, including: *Felis catus* (cat), *Canis familiaris* (dog), *Bos taurus* (cow), *Equus caballus* (horse), *Sus domesticus* (pig), *Ovis aries* (sheep) and *Gallus gallus* (chicken – cecum) (Table 1). Multiple independent animals were sampled for each species. After isolation, crypts were cultured in either 50% L-WRN CM or media lacking the WRN factors (D10). The majority of crypt samples cultured in 50% L-WRN CM expanded and grew for several passages and lines could be maintained in 50% L-WRN CM to high passage number over long periods of time (Table 1), whereas none of the isolated crypts cultured in D10 grew to the first passage (Fig. 2A).

**Table 1.**
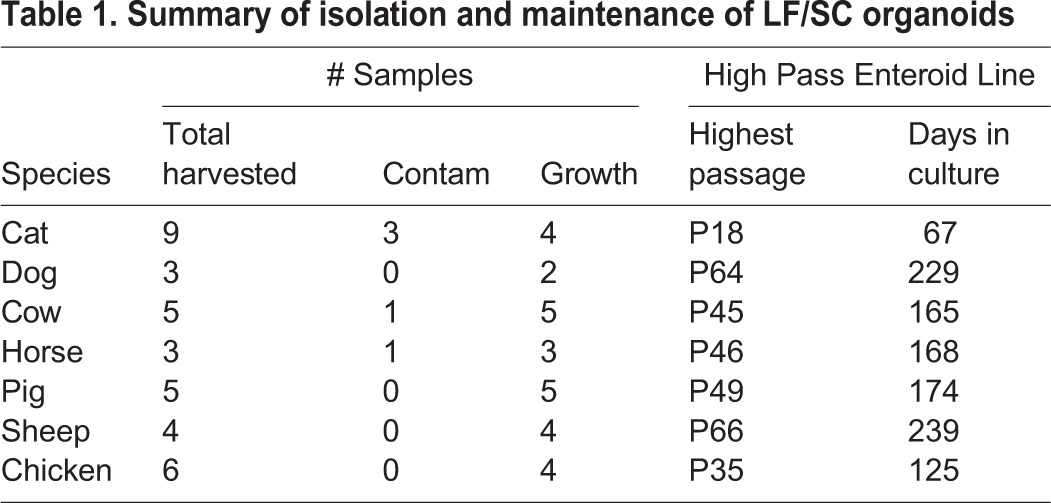
**Summary of isolation and maintenance of LF/SC organoids**

Small enteroids could be observed in 50% L-WRN CM within the first day after isolation and increased in number and size by the end of the first week (Fig. 2A). Enteroids from all LF/SC animals expanded and many began to obtain crypt-like folds with subsequent passages (Fig. 2A, P5 and P>10). High passage enteroid lines were maintained at high density (Fig. 2A, P>10, 500 µm). Like the mouse enteroids, LF/SC enteroids failed to grow after isolation in D10 media or after passage to 50% L CM when initially grown in 50% L-WRN CM to high passage (Fig. 2A), demonstrating the requirement for the WRN factors to stimulate enteroid growth as D10 and 50% L CM lack the WRN factors. Additionally, LF/SC enteroids can be cryopreserved, thawed, and expanded, providing a banked resource for continued experimentation (Fig. S2). The LF/SC enteroid passage history demonstrates the growth potential of these lines, where the cumulative fold expansion increased over time as enteroids were passed from well to well at high density (Fig. 2B). Likewise, the number of enteroids increased for high passage LF/SC lines when they were passed at a ratio of 1:2 over two passages (Fig. 2C).

**Fig. 3.**
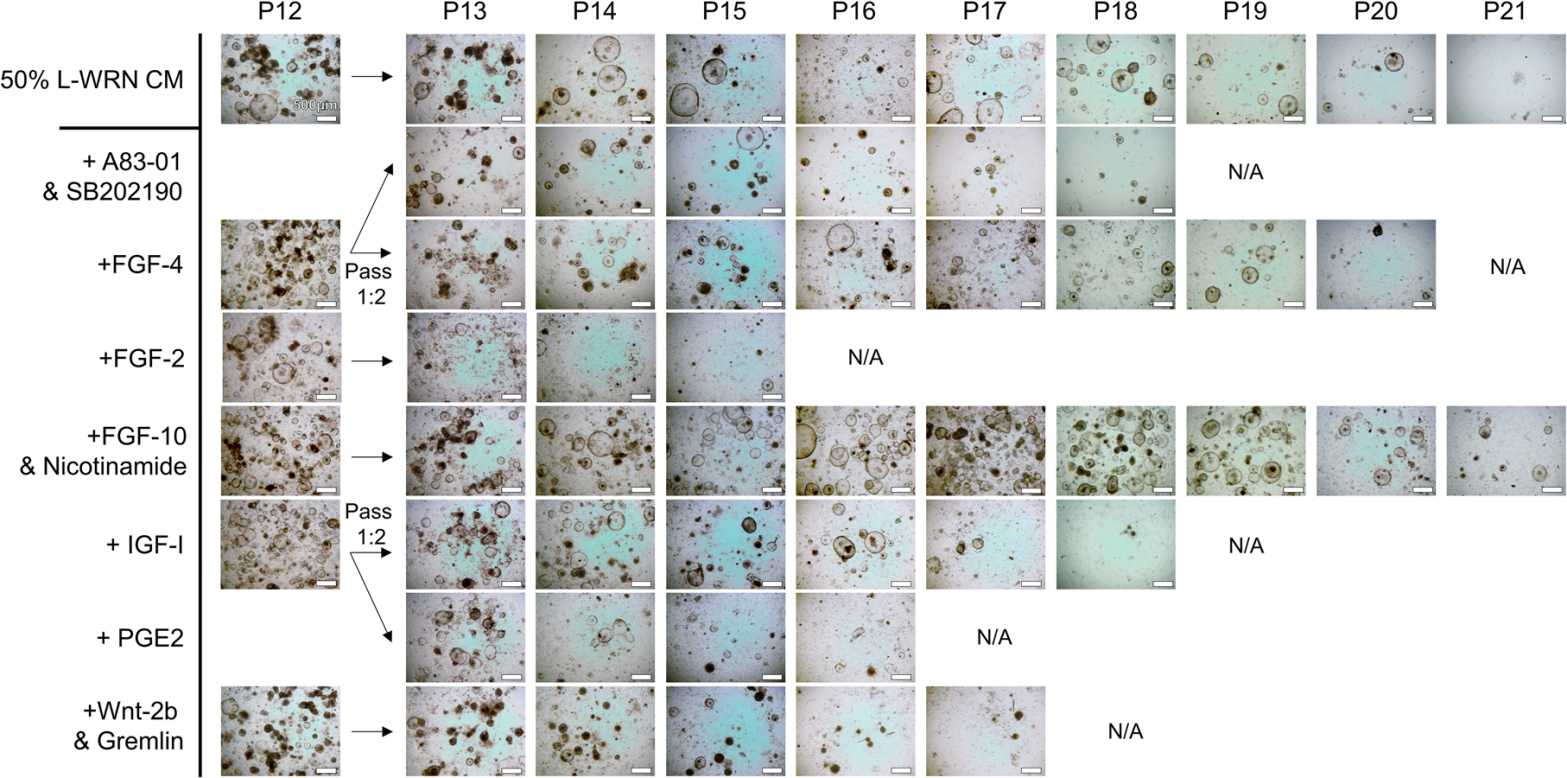
**Supplementation of 50% L-WRN CM with crypt niche factors.** Cat enteroids were passed to multiple wells at P12 and cultured with 50% L-WRN CM or with additional crypt niche factor(s) shown to modulate organoid growth in various platforms. The individual cultures were passed every 3-4 days and monitored for growth. Cultures were terminated when the number and growth of the enteroids dissipated (N/A). None of the crypt niche factors rescued the cat enteroids much beyond those grown with 50% L-WRN CM.

Although we have been able to grow one cat intestinal enteroid line to relatively high passage (P18) (Table 1), and have demonstrated the requirement of 50% L-WRN CM for cat enteroid growth (Fig. 2A), cat enteroids cease to expand around P10 and begin to growth arrest around P13-P18 (Fig. 3). We are not certain why this limited expansion is restricted to the cat samples. Enteroids from all other LF/SC species tested here continue to grow and can be maintained at high density well past P10. We did observe a mesenchymal-like cell type growing with the cat enteroids (Fig. 2A, P5) which wane in number around P7-P9 before the enteroids begin to growth arrest, suggesting these cell types are needed for cat enteroid growth. Indeed, culture systems for intestinal cell growth have been developed based on a requirement of co-culturing mesenchymal/stromal components with intestinal crypts (Lahar et al., 2011; Li et al., 2016; Ootani et al., 2009; Spence et al., 2011). In an attempt to rescue the cat enteroids, we supplemented 50% L-WRN CM with various factors that have been shown to induce enteroid growth in other platforms or to be secreted by mesenchymal/stromal cells within the crypt niche (Fig. 3) (Date and Sato, 2015; Roulis and Flavell, 2016). These factors included: ALK5 inhibitor A83-01 and p38 MAP kinase inhibitor SB202190 (Khalil et al., 2016; Sato et al., 2011a), human FGF-4 (Clevers, 2016; McCracken et al., 2014; Spence et al., 2011), human FGF-2 (FGF-basic) (Clevers, 2016), human FGF-10 and nicotinamide (Barker et al., 2010; Boj et al., 2015; Sato and Clevers, 2015), human IGF-I (Reynolds et al., 2014; Simmons et al., 2007), Prostaglandin E2 (PGE2) (Goessling et al., 2009; Jung et al., 2011; Pierzchalska et al., 2012), or mouse Wnt-2b and human Gremlin (Aoki et al., 2016; Stzepourginski et al., 2017). None of these conditions rescued the cat enteroids to significantly improve growth beyond that in 50% L-WRN CM alone (Fig. 3). We continue to test other conditions in an attempt to grow the cat enteroids to higher passage number.

### LF/SC enteroids express *LGR5* and contain proliferating crypt-like regions

**Fig. 4.**
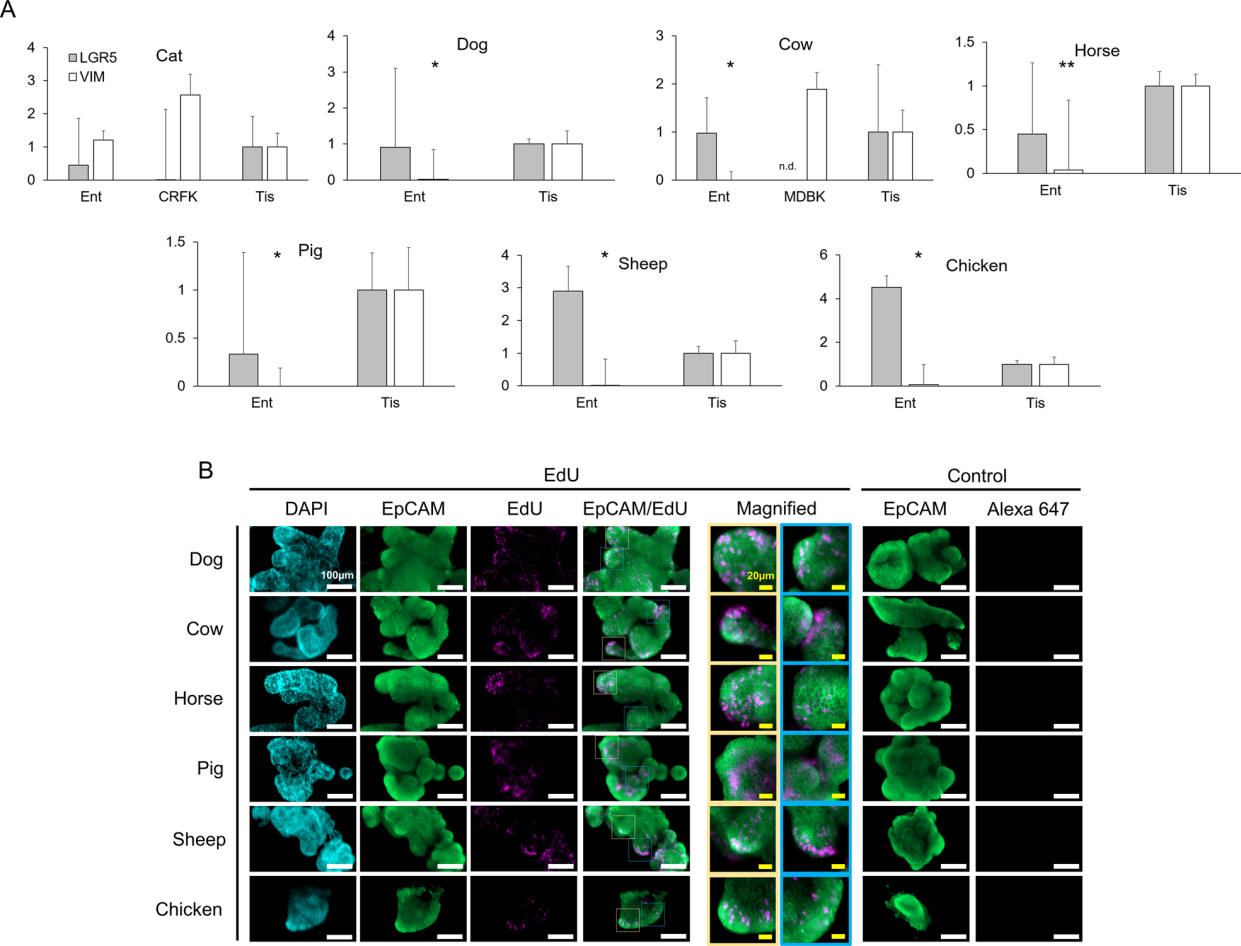
**LGR5 expression in LF/SC enteroids.** (A) *LGR5* RNA expression in LF/SC enteroids: cat (P11-12), dog (P27-34), cow (P18-27), horse (P29-35), pig (P48-49), sheep (P28-35), chicken (P18-28). qPCR of *LGR5* and *VIM* expression in enteroids (Ent) and control tissue (Tis), student's *t*-test: *P≤0.05, **P≤0.005. Most LF/SC enteroids express *LGR5* above the control tissue. LF/SC enteroids also have very low expression of mesenchymal marker VIM as compared to control tissue and kidney epithelial cells [cat (CRFK) and cow (MDBK)]. Enteroid values are fold change of control tissue; mean±se.m.; n.d., not detected; *n*=3. (B) EdU incorporation using the Click-iT EdU Alexa Fluor 647 assay in LF/SC enteroids confirms active proliferation with crypt-like regions. IFA of LF/SC enteroids probed with multi-species reactive α-EpCam (green, Alexa Fluor 488), Click-iT EdU (pink, Alexa Fluor 647), and nuclei stained with DAPI (blue). Merged EpCam and EdU images shows distinct regions of proliferation along crypt-like structures. Enteroids probed with α-EpCam (green) fail to stain pink (Alexa 647) in negative control IFAs lacking the Click-iT EdU.

The growth specificity of LF/SC enteroids in 50% L-WRN CM suggests the WRN factors are sufficient to sustain LGR5^+^ stem cells within the LF/SC enteroids, providing for their propagation, thus increasing enteroid size and cell number over time (Fig. 2). We harvested RNA from high passage LF/SC enteroids to determine the expression of stem cell marker *LGR5* by qPCR, using whole intestinal tissue as a control that was collected and cryopreserved at the time of crypt harvest. The control tissue represents the various cell types of the intestine, containing tissues of the serosa, muscularis, submucosa, and mucosa. Enteroids from dog, cow, sheep and chicken all expressed *LGR5* at or above the control tissue (Fig. 4A). Although lower than control tissue, LGR5 expression was also detected in the cat, horse and pig samples suggesting the presence of LGR5^+^ cells in these enteroids as well. *LGR5* was low or not detected in non-stem cell control kidney epithelial cells from cat (CRFK) and cow (MDBK), respectively. We also determined the expression of mesenchymal marker vimentin (*VIM*) in the LF/SC enteroids (Fig. 4A). As expected, *VIM* expression in the two control kidney lines CRFK and MDBK was robust. In contrast to *LGR5* expression, *VIM* expression was significantly lower than control tissue in the dog, cow, horse, pig, sheep and chicken enteroids. The low expression of VIM in these samples suggests they are able to grow in 50% L-WRN CM without the presence of mesenchymal cells (Sato et al., 2009; VanDussen et al., 2015). *VIM* was expressed above the control tissue in the cat enteroid line, further supporting the possibility that mesenchymal cells are present and required for the growth and expansion of cat enteroids.

LF/SC enteroids are a collection of continuously spaced cells (Fig. 4B, DAPI) with morphological characteristics similar to enteroids from mouse and human samples (Fig. 1C) (Date and Sato, 2015; Mahe et al., 2013; Sato et al., 2011a, 2009), containing budding structures producing invaginations and folds reminiscent of crypt-villus units (Fig. 4B). In addition, brief labeling with EdU revealed distinct regions of cell proliferation (Fig. 4B), many of which are in areas exhibiting crypt-like budding and folding.

We show that culturing methods for growing intestinal crypt-derived mouse and human enteroids are applicable when applied to a number of animals of veterinary relevance. LF/SC enteroids grow and expand when cultured with 50% L-WRN CM, can be maintained to high passage number, and can be cryopreserved allowing for their use as resources for downstream experimentation. These cell lines offer additional models for the study of gastrointestinal development and disease that are pertinent to both veterinary and human medicine. In addition, these cell lines can be used to study host-pathogen interactions of zoonotic enteric pathogens and the intestinal cells with which they associate. Although we have not tested the individual requirements of Wnt3a, R-spondin-3 and Noggin in this study, this could be pursued in the future using cell lines that make conditioned media for each of these factors separately (Ettayebi et al., 2016; Heijmans et al., 2013). For example, in some platforms mouse small-intestinal enteroids can be cultured with media cocktails that contain just R-spondin and Noggin because Paneth cells within enteroids secrete Wnt3 (Sato et al., 2011b, 2009). The L-WRN conditioned media used here is a relatively simple, inexpensive, robust and readily attainable culture platform compared to other alternatives. The successful culturing of LF/SC enteroids promises to increase the accessibility of these studies to laboratories not associated with facilities compatible with LF/SC animals. Furthermore, it will decrease the number of animals and the associated housing costs required to conduct gastrointestinal related research using LF/SC organisms.

## MATERIALS AND METHODS

### Media and L-WRN cell line

D10 media is DMEM high glucose (Thermo Fisher, Waltham, MA, USA, Cat# 12800) supplemented with 10% FBS (VWR Seradigm Premium Grade). The L (CRL-2648) and L-WRN (CRL-3276) cell lines were obtained from American Type Culture Collection (ATCC). The 50% L-WRN conditioned media (50% L-WRN CM) was made as previously described (Miyoshi and Stappenbeck, 2013) using the L-WRN cell line, which was derived from the L cell line (CRL-2648). The L cell conditioned media is media incubated with the L cell line (CRL-2648) which lacks the WRN factors, thus 50% L cell conditioned media (50% L CM).

### Animals

All Louisiana State University (LSU) Institutional Animal Care and Use Committee (IACUC) protocols were followed for mouse tissue collection. All LSU IACUC guidelines were followed for intestinal tissue collection from LF/SC animal carcasses at local processing and shelter facilities, such as not handling live animals or in any way being involved in the euthanasia schedule.

### Protein alignments

The human and mouse protein sequence of Wnt3a, R-spondin, and Noggin were used find the LF/SC orthologs using NCBI HomoloGene (https://www.ncbi.nlm.nih.gov/homologene) and/or NCBI BLAST (https://blast.ncbi.nlm.nih.gov/Blast.cgi) against the appropriate genome. The following protein sequences were obtained from NCBI and used to make alignments in UGENE (Okonechnikov et al., 2012) using the Kalign algorithm (Edgar, 2004); Wnt3a: human (NP_149122.1), mouse (NP_033548.1), cat (XP_003980679.1), dog (XP_539327.3), cow (XP_015327344.1), horse (XP_014595070.1), pig (XP_003123669.1), goat (XP_013820403.1), sheep (XP_012026443.1), chicken (NP_001165072.1); R-spondin-3: human (NP_116173.2), mouse (NP_082627.3), cat (XP_003986583.1), dog (XP_005615677.2), cow (NP_001069502.1), horse (ABV31708.1), pig (XP_001926731.4), goat (XP_005684536.1), sheep (XP_004011185.1), chicken (XP_004940314.1); Noggin: human (NP_005441.1), mouse (NP_032737.1), cat (XP_011278221.1 and XP_011278233.1), dog (BAJ24018.1), cow (XP_002695600.1), horse (AAM34779.1), pig (NP_001137163.1), goat (XP_013827246.1), sheep (NP_001167581.1), chicken (AAC83570.1).

### Crypt isolation and culturing of enteroids

Murine intestinal crypts were harvested from a 6 week old female BALB/c mouse to test the viability of the 50% L-WRN CM made from the L-WRN cell line. To obtain LF/SC intestine samples, we established working relationships with local animal processing and shelter facilities that routinely euthanize animals. Samples were taken from the terminal ileum of mammal and the cecum of chicken carcasses. Sampling was conducted within 30 min after euthanasia and samples were transported in phosphate buffered saline (PBS) on ice. Several 1 cm^2^ pieces of the intestine were transferred to cryovials and flash frozen in LN_2_ to bank control tissue for use in qPCR. Harvesting and isolation of crypts followed previously described protocols (Miyoshi and Stappenbeck, 2013) with minor modification. Briefly, tissue was cut longitudinally, trimmed to 1 cm^2^ pieces, rinsed with PBS, placed in D10, villi were removed by scraping, tissue was minced, digested with collagenase (Sigma-Aldrich, St. Louis, MO, USA, Cat # C0130) at 37°C for 1-1.5 h while vortexing every 10-15 min, crypts were filtered with a 70 µm cell strainer, rinsed with D10 three times, and after final centrifugation suspended in 20 µl Matrigel matrix (Corning, Corning, NY, USA, Cat # 354234) per well and seeded to a 24-well plate. Crypts were then cultured and enteroids maintained in 50% L-WRN CM with 10 µM Y-27632 ROCK inhibitor (BioVision, Milpitas, CA, USA, Cat # 1596) and 10 µM SB-431542 TGF-β inhibitor (BioVision, Cat # 1674). High passage enteroid lines were confirmed by sequencing of the GAPDH gene and were monitored for contamination.

### Maintenance of enteroids

Enteroids were passed every 3-4 days, as previously described (Miyoshi and Stappenbeck, 2013) with minor modification. Briefly, Matrigel was washed with PBS, digested with 2.5× trypsin at 37°C for 5 min, enteroids were rinsed in D10, incubated in D10 with 10 µg/ml muramyl dipeptide (Sigma-Aldrich, Cat # A9519) for 10 min at room temperature which has been shown to increase LGR5^+^ stem cell growth (Nigro et al., 2014), then centrifuged (200 rcf), suspended in 20 µl Matrigel per well, seeded to a 24-well plate and grown in 50% L-WRN CM with 10 µM Y-27632 and 10 µM SB-431542.

Additional factors added to 50% L-WRN CM with 10 µM Y-27632 and 10 µM SB-431542 in an attempt to rescue the cat enteroids include: A83-01 (10 µM; BioVision, Cat # 1725-1), SB202190 (10 µM; AdipoGen, San Diego, CA, USA, Cat # AGCR10028M001), human FGF-4 (500 ng/ml; Peprotech, Rocky Hill, NJ, USA, Cat # 100-31), human FGF-2 (FGF-basic) (100 ng/ml; Peprotech, Cat # 100-18B), human FGF-10 (100 ng/ml; Peprotech, Cat # 100-26), nicotinamide (100 µM; Alfa Aesar, Tewksbury, MA, USA, Cat # A15970-22), human IGF-I (100 ng/ml; Peprotech, Cat # 100-11), PGE2 (5 µg/ml; TCI America, Portland, OR, USA, Cat # P1884-1MG), mouse Wnt-2b (100 ng/ml; R&D Systems, Minneapolis, MN, USA, Cat # 3900-WN/CF), human Gremlin (200 ng/ml; Peprotech, Cat # 120-42).

To cryo-preserve the cell lines, Matrigel with enteroids was scrapped with a pipette tip, centrifuged (200 rcf), suspended in D10 with 50% FBS, then mixed with an equal amount of D10 with 20% DMSO, and transferred to a cryovial. Vials were placed in a CoolCell container (Corning) at −80°C for 24 h then transferred to LN_2_ storage.

### Protein alignments

Protein sequences were aligned in UGENE (Okonechnikov et al., 2012). Details and accession numbers are provided in the Supplementary Materials.

### Microscopy

Brightfield images of enteroid in Matrigel were taken with an Olympus IX71 microscope, Olympus U-CMAD3 camera (10×) or a Leica DMIRB microscope, Olympus DP80 camera (2.5×) using the cellSens software (Olympus).

### Enteroid counts

Fold expansion of enteroids over time was determined by recording the ratio at which wells were split at each passage throughout the passage history of that cell line, for example two wells of enteroids passed to four new wells (1:2).

To determine enteroid expansion during passage, 200 high passage (*P*>10) enteroids were seeded in Matrigel matrix to two wells (P1). After 3-4 days of growth the enteroids were counted and passed (P2), split 1:2. Again, after 3-4 days of growth the enteroids were counted.

### RNA isolation and qPCR

RNA from enteroids, control tissue, and kidney epithelial cells (CRFK and MDBK) was isolated using the RNeasy Plus mini kit (Qiagen) according to the manufacturer's protocol, with DNase treatment (Qiagen) on the tissue controls samples. The High Capacity RNA-to-cDNA kit (Thermo Fisher 4387406) was used to obtain cDNA from 500 ng of RNA. qPCR negative controls omitted the RT enzyme. qPCR was performed with the PowerUp™ SYBR™ Green Master Mix (Thermo Fisher A25742) with a final primer concentration of 0.5 µM (Table S1) and 10 ng of cDNA, and run on an Applied Biosystems (ABI) 7900HT Sequence Detection System. Data are the average of three independent cDNA samples and technical replicates conducted for each sample per qPCR run. Data were normalized to sample GAPDH values, control tissue values were set to 1 and enteroid values expressed as a ratio to control tissue using the 2-ΔΔCt method (Livak and Schmittgen, 2001). Raw data for three independent cDNA samples and their technical replicates were analyzed in ExpressionSuite Software v 1.0.3 (Thermo Fisher). Biological groups were designated by sample type, with control tissue selected as the biological reference group, and data were normalized using GAPDH as an endogenous control. One standard deviation was used to determine the RQ minimum and maximum (error bars).

### Immunofluorescence assays

Enteroids were suspended in 25 µl of 50% Matrigel diluted in PBS, plated as a thin mound in an 8-well chamber slide (Corning), and cultured in 200 µl 50% L-WRN CM at 37°C for two days. Cultures were rinsed with PBS prior to fixation and between incubations steps. Click-iT EdU Alexa Fluor 647 assay (Thermo Fisher, Cat # C10640) was used to label proliferating cells within the enteroids according to the manufactures protocol and O'Rourke et al. (2016). Briefly, enteroids were incubated for 2 h with EdU, fixed in 4% formaldehyde, permeabilized with 0.5% Triton X-100, incubated with 50 mM NH_4_Cl in PBS (Mahe et al., 2013), blocked in 1% BSA, processed with Click-iT chemistry to label with Alexa Fluor 647, labeled with 1:400 primary mouse α-EpCAM (Sigma-Aldrich, Cat # SAB4200473) in 1% BSA at 4°C O/N, then 1:250 secondary goat α-mouse Alexa Fluor 488 (Thermo Fisher) in 1% BSA at 4°C for 3 h, nuclei were stained with DAPI (1 µg/ml) for 10 min, and slides were mounted with ProLong Diamond (Thermo Fisher). Images were taken on a Zeiss Observer.Z1 Axio microscope with z-stack layering and processed with the ‘best fit’ option to reduce background, slight gamma value adjustment, then deconvolution and extended depth of focus. The negative control images were captured and processed the same as the comparable IFA.
